# Task shifting redefined: removing social and structural barriers to improve delivery of HIV services for people who inject drugs

**DOI:** 10.1186/1477-7517-10-20

**Published:** 2013-10-04

**Authors:** Lianping Ti, Thomas Kerr

**Affiliations:** 1 British Columbia Centre for Excellence in HIV/AIDS, St. Paul’s Hospital, 608-1081 Burrard Street, Vancouver, BC V6Z 1Y6, Canada; 2 Department of Medicine, St. Paul’s Hospital, University of British Columbia, 608-1081 Burrard Street, Vancouver, BC V6Z 1Y6, Canada

**Keywords:** Task shifting, People who inject drugs, Stigma and discrimination, HIV services

## Abstract

HIV infection among people who inject drugs (IDU) remains a major global public health challenge. However, among IDU, access to essential HIV-related services remains unacceptably low, especially in settings where stigma, discrimination, and criminalization exist. These ongoing problems account for a significant amount of preventable morbidity and mortality within this population, and indicate the need for novel approaches to HIV program delivery for IDU. Task shifting is a concept that has been applied successfully in African settings as a way to address health worker shortages. However, to date, this concept has not been applied as a means of addressing the social and structural barriers to HIV prevention and treatment experienced by IDU. Given the growing evidence demonstrating the effectiveness of IDU-run programs in increasing access to healthcare, the time has come to extend the notion of task shifting and apply it in settings where stigma, discrimination, and criminalization continue to pose significant barriers to HIV program access for IDU. By involving IDU more directly in the delivery of HIV programs, task shifting may serve to foster a new era in the response to HIV/AIDS among IDU.

## 

The HIV/AIDS epidemic among people who inject drugs (IDU) remains a major public health challenge globally. This epidemic persists despite the fact that a range of effective preventive interventions and treatments exist [1]. A review by the Reference Group to the UN on HIV and Injecting Drug Use concluded that access to HIV prevention, treatment and care among IDU globally was extremely low and that urgent action was needed to rectify this situation [2]. Various factors contributing to low access to HIV prevention and treatment among IDU have been identified, including the unavailability of programs, as well as an overreliance on drug law enforcement, incarceration, and mandatory drug detention programs as primary responses to the harms of injection drug use [3]–[6].

A growing body of literature highlights a range of downstream social and behavioural impacts of the global emphasis on drug law enforcement. For example, fear of confrontations with police perpetuates unwillingness among IDU to access essential HIV-related services [7,8]. The emphasis on punishment of IDU for their behaviours also fuels negative public opinion of this population, including among healthcare workers, which makes ensuring access to prevention and treatment of HIV challenging if not impossible [8,9]. For example, throughout the Asia-Pacific region, many IDU encounter delays in the provision of healthcare services, refusal of treatment by healthcare workers, as well as breaches of confidentiality, including sharing of information between healthcare workers and police [10,11]. In some settings, individuals are registered as drug users within national or regional databases upon seeking care or treatment [8]. Negative physician attitudes towards HIV-positive IDU have also led to suboptimal ART treatment and care for this subpopulation due to concerns over non-adherence and antiretroviral resistance [11]–[13]. Pervasive stigma and the associated self-imposed isolation that often results can also render individuals reluctant to access services due to fears that family, community members, and employers may shun them for their drug using behaviours [14]–[16]. Collectively, these barriers to HIV prevention and treatment highlight the urgent need for novel methods of healthcare delivery for this population.

One concept that has been applied to improve the delivery of HIV services in African settings is that of task shifting. Defined as the systematic delegation of tasks from specialized cadres to cadres with less training such as nurses or lay workers, task shifting has been used as an effective strategy to address the current healthcare worker shortage in many African countries [17]–[20]. A body of literature supports the use of task shifting as a successful approach in delivering healthcare services including HIV testing, counseling, and ART treatment by lay workers [21]–[24]. In addition to the success of this model among heterosexual populations within resource limited settings, a systematic review has revealed that task shifting can also be applied effectively to more marginalized populations, including men who have sex with men [25]. In addition to the role of task shifting as a means to improve coverage of HIV services, this concept has also been applied to alleviate the economic burden imposed on many developing countries [18]. Due to recent financial cutbacks made by the Global Fund to Fight AIDS, Tuberculosis and Malaria, the organization is now considerably more limited in its ability to respond to the global HIV pandemic [26,27]. In a time when resources are scarce, task shifting may help to relieve this situation and attempt to avert the HIV/AIDS crisis.

In light of the ongoing problems in ensuring access to essential HIV prevention and treatment services for IDU, there may be an opportunity to reconceptualize task shifting as a way of overcoming social and structural barriers to HIV-related services. A large body of evidence indicates that peer-run initiatives can extend the reach and effectiveness of conventional public health programs by reaching high-risk IDU [28]–[30]. Accordingly, the WHO, UNODC, UNAIDS Technical Guide recommend community-based outreach methods as an essential approach for service delivery [1]. However, the involvement of IDU in providing HIV services need not be limited to those efforts that aim to extend the reach of existing programs and may have value in other areas. Shifting HIV services from professional healthcare workers to peers may also serve to address the existing stigma that IDU experience within healthcare settings, thereby improving access to these services, especially in the Asia-Pacific region where the annual prevalence of HIV testing among IDU is as low as 20% [31]. By creating peer-involved HIV testing clinics and pairing physicians with peers, IDU may be more likely to use these services without fear of being discriminated by healthcare workers or fear of being registered as drug users within official registries. Indeed, past research has shown that drug-user led interventions are more acceptable to IDU than conventional public health programs,[32]–[34] and that this is due in part to perceived acceptance of their drug use behaviors by their peers [28,29]. In this sense, there may also be potential for peer-delivered HIV services that do not involve healthcare professionals, as many IDU may prefer to have their peers deliver these services to avoid frequent interactions with healthcare workers.

Additionally, task shifting may avert some problems caused by police in countries with a heavy reliance on law enforcement. By shifting delivery of care from healthcare professionals to peers, or by incorporating peer workers into professionally-led services, a reduction in stigma and discrimination in these settings may be achieved [32]. Likewise, this type of shift in service delivery may address some concerns among IDU about information sharing between public health systems and enforcement officials.

While there is potential for task shifting to reduce stigma and discrimination in these settings and thus provide greater coverage of HIV prevention and treatment services, it is important to recognize the political barriers that may restrict the wide implementation of these programs; particularly, the lack of governmental and public support for harm reduction programs. Therefore, in order for task shifting to be successfully and sustainably implemented within these settings, there is still a need to shift public and policy thinking towards harm reduction practices through the collective involvement of the community, researchers, service providers, advocates, and policy makers.

HIV/AIDS among IDU has taken a massive toll in terms of human suffering and economic impacts in countries throughout the world. High rates of preventable HIV infection, HIV-related morbidity and mortality among IDU, as well as increasing expenditures on HIV-related care and treatment services are major consequences of suboptimal HIV prevention and treatment. There is now an obvious need for innovation in the delivery of programs and services for IDU. Given the evidence indicating positive benefits of peer-led interventions for IDU, as well as the success of task shifting in settings with human health resource shortages, shifting the delivery of conventional HIV/AIDS programs and services to IDU themselves may serve to address the severe stigmatization and discrimination that characterizes the existing healthcare context in many settings hard hit by IDU-driven HIV epidemics. In turn, this novel approach to task shifting may foster a new era in the response to HIV among IDU.

## Competing interests

The authors declare that they have no competing interests.

## Authors’ contributions

The specific contributions of each author are as follows: LT prepared the first draft of the manuscript; TK provided critical comments on the first draft of the manuscript; Both authors approved the final version to be submitted.
